# Immune-Mediated Pathogenesis in Dengue Virus Infection

**DOI:** 10.3390/v14112575

**Published:** 2022-11-21

**Authors:** Arshi Khanam, Hector Gutiérrez-Barbosa, Kirsten E. Lyke, Joel V. Chua

**Affiliations:** 1 Division of Clinical Care and Research, Institute of Human Virology, University of Maryland School of Medicine, Baltimore, MD 21201, USA; 2 Center for Vaccine Development and Global Health, University of Maryland School of Medicine, Baltimore, MD 21201, USA

**Keywords:** dengue virus, host immune response, inflammation, exosomes, immunopathogenesis

## Abstract

Dengue virus (DENV) infection is one of the major public health concerns around the globe, especially in the tropical regions of the world that contribute to 75% percent of dengue cases. While the majority of DENV infections are mild or asymptomatic, approximately 5% of the cases develop a severe form of the disease that is mainly attributed to sequential infection with different DENV serotypes. The severity of dengue depends on many immunopathogenic mechanisms involving both viral and host factors. Emerging evidence implicates an impaired immune response as contributing to disease progression and severity by restricting viral clearance and inducing severe inflammation, subsequently leading to dengue hemorrhagic fever and dengue shock syndrome. Moreover, the ability of DENV to infect a wide variety of immune cells, including monocytes, macrophages, dendritic cells, mast cells, and T and B cells, further dysregulates the antiviral functions of these cells, resulting in viral dissemination. Although several risk factors associated with disease progression have been proposed, gaps persist in the understanding of the disease pathogenesis and further investigations are warranted. In this review, we discuss known mechanisms of DENV-mediated immunopathogenesis and its association with disease progression and severity.

## 1. Introduction

Dengue, caused by the dengue virus (DENV), is one of the most prevalent tropical infectious diseases affecting close to 400 million people worldwide annually [1]. Although the majority of people infected with DENV are either asymptomatic or have mild flu-like symptoms, some develop severe disease, such as dengue hemorrhagic fever (DHF) and dengue shock syndrome (DSS), marked by presence of coagulation abnormalities, capillary leakage, and increased vascular fragility [2,3]. Secondary DENV infection has been associated with severe dengue infection [4]. Dengue typically presents with fever, nausea, muscle and joint pain, and headache. Atypical presentations include dermatologic manifestations with morbilliform rashes and/or petechiae [5]. Clinical presentations and severity are dependent on several factors, including the virus, epidemiology, host immune response, and genetic makeup [6]. Currently, treatment involves only the supportive measures as there is no specific antiviral therapy approved to treat or prevent dengue infection.

The dengue virus is a single-stranded positive-sense RNA flavivirus enclosed within the capsid and encircled by an envelope protein comprising E (envelope) and prM/M (premembrane/membrane) structural proteins. Nonstructural proteins (NS) include NS1, NS2A, NS2B, NS3, NS4A, NS4B, and NS5. Both the structural and nonstructural proteins are transcribed and translated during the viral replication cycle, making them available to intracellular antigen-processing pathways. These NS proteins have different functions. For instance, NS1 interacts with NS4A/B to promote viral replication, whereas NS3 performs helicase and protease functions [7,8]. NS4A instigates autophagy, while NS4B promotes dissociation of NS3 helicase from viral RNA [9,10]. NS5B remains the largest and the most conserved DENV protein, functioning as both an RNA-dependent RNA polymerase that replicates the viral RNA and an RNA methyltransferase enzyme that protects the viral genome by RNA capping, facilitating polyprotein translation [11]. Given that NS5 is crucial for viral replication and serves as a major target for cytotoxic T-cell responses [12], much attention is being given to target it for the vaccine development and antiviral therapies. Generally, NS proteins are responsible for host immune evasion by inhibiting the induction of different signaling pathways in the infected cells [13]. DENV can block pattern recognition receptor signaling and type 1 interferon response by targeting retinoic acid-inducible gene-I (RIG-I)-like receptors (RLR), nucleotide-binding oligomerization domain (NOD)-like receptors, and toll-like receptors (TLRs) [14,15]. Methylation of NS5 2′-O of 5′ inhibits the virus from being sensed by RIG-I, while NS3 is involved in blocking RIG-I translocation to mitochondria by binding with the mitochondrial-targeting chaperone protein 14-3-3ϵ [16]. NS4A is also associated with the inhibition of RIG-I interaction with mitochondrial antiviral-signaling protein (MAVS) by binding to the MAVS CARD-like domain and transmembrane domain [17]. Similarly, NS2A and NS4B are involved in blocking the RIG-I/MAVS signaling pathway by shutting down the phosphorylation of TANK binding kinase1 (TBK1) and interferon regulatory factors (IRFs), inhibiting IFNβ induction [18,19]. NS2B/3 inhibits interferon production by cleaving the stimulator of interferon genes protein [20].

There are four major serotypes, namely DENV-1, 2, 3, and 4 [21]. DENV infection occurs after a bite of a female Aedes mosquitoes (Aedes aegypti and less commonly cold-adapted Aedes albopictus). Initial infection with any of the DENV serotypes provides imperfect protection against all four serotypes due to crossreactivity; this cross-reactive immunological memory appears to be short-lived. After neutralization of the immunological memory, protective response can be achieved if the patient gets reinfected with the same serotype. Subsequent infection with different serotypes is associated with severe forms of the disease and imposes serious complications, including the risk of mortality [22]. All the serotypes of DENV are capable of causing disease; therefore, developing an effective DENV vaccine is quite challenging, as the vaccine is required to be highly effective against all four serotypes. Several DENV vaccine candidates are under clinical evaluation, of which CYD-TDV (Dengvaxia^®^) has been recommended by World Health Organization for individuals aged 9–45 years living in endemic areas and with confirmed previous DENV infection, and has been licensed in 20 countries; however, serious safety concerns have been raised [23]. In fact, CYD-TDV enhances the risk of severe DENV infection, as defined by hospitalization, in vaccinated individuals who were not previously exposed to DENV prior to vaccination [24]. Therefore, an extensive understanding of immunological correlates of protection against DENV infection is the prerequisite for the development of effective DENV vaccines.

The pathogenesis of DENV infection is attributed to the complex interplay between the virus, host genes, and host immune response, with the host immune response critically involved in the pathogenesis of DENV infection. In fact, DENV infection manifests as the severe form of the disease when the infection is being eliminated by the host immune response and is not correlative with peak viral load [25]. Moreover, antibody-dependent enhancement (ADE) upon infection also contributes to pathogenesis and virulence of the disease [26]. Antibody-dependent enhancement develops after the antibodies from a previous heterotypic infection fail to neutralize a secondary infection with a distinct subtype, even after binding with the viral proteins [27]. The virus–antibody complex is then phagocytosed by the cells via crystallizable fragment-γ (Fcγ) receptors and results in increased viremia and pathology [28]. Since the pathogenesis of DENV infection is extremely complex, it requires further investigations. In this review, we will discuss the immunological mechanisms associated with the pathophysiology of DENV infection.

## 2. Involvement of T Cells in the Pathogenesis of DENV Infection

**Figure 1:**
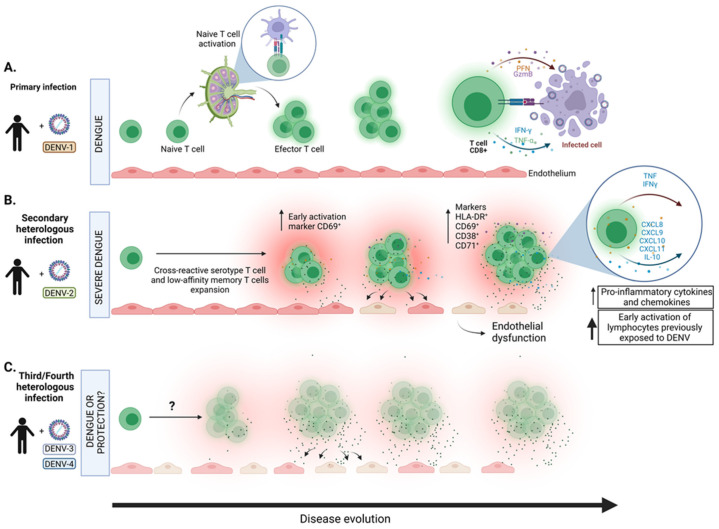
T cell response during DENV infection. History of exposure to dengue virus (DENV) is one of the factors that drive the T cell response in the evolution of the disease. (**A**) During primary infection, naïve T cells differentiate into effector T cells, leading to clearance of infection by direct lysis or by releasing antiviral cytokines, such as IFN-γ and TNF-α. (**B**) During a secondary heterologous infection, a robust immune response is developed by the early activation of cross-reactive serotype T cells, which produce abundant proinflammatory cytokines and chemokines, leading to excessive inflammatory environment, causing endothelial dysfunction that could trigger vascular permeability. (**C**) During a third or fourth heterologous infection, the activation of cross-reactive serotype T cells and their role in the severity or protection against infection is largely unknown.

As the DENV infection caused by different serotypes and long-term cross-serotype protective immunity is lacking following primary infection, individuals in dengue endemic areas frequently acquire symptomatic infection more than once [29]. While CD8 T cells have critical functions in neutralizing harmful viruses, they have also been implicated in the pathogenesis of DENV infection. Severe dengue manifestations occur 10- to 20-fold higher in patients with secondary DENV infection, suggesting that the priming of the adaptive immune system to one DENV serotype enhances the risk of severity during secondary infection with different serotypes [6]. Furthermore, severe symptoms such as vascular leak, coagulopathy, and cytokine storm occur prominently when viremia is rapidly declining. Primary DENV infection induces the activation of naïve CD8 T cells to differentiate into effector T cells, which will clear the infection either directly by lysing the virus-infected cells or by producing cytokines [29]. The presence of diverse HLA-restricted T-cell epitopes on different proteins is seen in individuals who are immune to DENV infection. The identification of DENV’s structural and nonstructural proteins is mediated by CD4 and CD8 T cells, respectively [30,31]. T cells can respond to secondary infection caused by a different DENV serotype based on the epitope recognized [32]. Typically, a robust immune response develops in individuals who were previously exposed to the same serotype, whereas a cross-reactive serotype T-cell response develops after secondary infection. Increased frequencies of DENV-specific T cells were observed in acute DENV infection, and these cells represented activated phenotypes [33]. After the onset of infection, DENV-specific T cells expressed early activation marker CD69, and later displayed other activation markers such as CD38, CD71 and HLA-DR [34]. Investigation of HLA-DR^+^CD38^+^CD8^+^ T cells obtained from DENV-infected patients indicated the upregulated expression of those genes that are involved in T cell activation, proliferation, migration, and cytotoxicity. In addition, HLA-DR^+^CD38^+^CD8^+^ T cells display a higher expression of multiple inhibitory receptors that are involved in T cell receptor (TCR) signaling. Analysis of HLA-DR^+^CD38^+^ and HLA-DR^-^CD38^+^ effector CD8 T cells in dengue-infected patients from India and Thailand displayed the expansion of both the subsets in these patients, while the effector qualities were more prominent in HLA-DR^+^CD38^+^ CD8 T cells and were mostly directed against NS3 [35]. However, only a small fraction of effector CD8 T cells were producing IFN-γ upon stimulation with virus peptide pools, due to the downregulation of TCR signaling molecules. During primary DENV infection, homotypic T cells are generated against the infecting DENV serotype that correlates with lifelong protection against that serotype. However, secondary DENV infection with different serotypes leads to the development of nonprotective, cross-reactive T cells, resulting in ineffective viral control during secondary heterologous infection, and supports severe dengue disease by induction and excessive inflammatory cytokine production [33]. In fact, cytokine storms are seen during peak symptoms, whereby high levels of proinflammatory cytokines, such as tumor necrosis factor (TNF), soluble TNF receptor 1 (sTNFR1), sTNFR2, and interferon-γ (IFN-γ), and chemokines such as CXCL8, CXCL9, CXCL10, CXCL11, and CCL5, along with anti-inflammatory cytokine interleukin-10 (IL-10), were observed [36,37]. This enhanced cytokine response could trigger vascular permeability, which is resolved after patient recovery. Moreover, the presence of soluble markers of T cell activation, including sIL-2R, sTNFR, and sCD8, indicate systemic T cell activation. Indeed, the soluble factors were higher in patients with severe disease compared to those who had a mild form of the disease, which further confirms immune-mediated pathogenesis in DENV infection [38]. Development of DENV-specific cross-reactive CD8 T cells occurs following both primary and secondary infection. However, these cells do not correlate with the disease severity. Further, it was revealed that a higher polyfunctional CD8 T cell response is associated with HLA alleles, which are related to the reduced risk of severe dengue disease [33]. This is in accordance with the study showing higher frequencies of cytokine-producing DENV-specific CD8 T cells in children who develop subclinical secondary infection than those with symptomatic secondary infection [39]. DENV-specific IFN-γ-producing CD8 T cells are mainly effector memory cells (T_EM_) or effector memory cells re-expressing CD45RA (T_EMRA_) [40]. Transcriptomic profile of T_EM_ and T_EMRA_ cells revealed that these cells exhibit specialized gene expression profiles related to activation, co-stimulation, and effector function [41]. Analysis of DENV-specific T cells revealed differences between mild and severe DENV infection that may be associated with disease severity. During mild DENV infection, CD8 T cells possess cytolytic function, but cytokine-producing abilities are compromised. Conversely, with severe primary and secondary DENV infection, CD8 T cells exhibit higher IFN-γ and TNF-α cytokine responses, but cytolytic activity is impaired, potentially affecting viral control and augmenting immunopathology. DENV-specific CD8 T cells express inhibitory receptor-programmed death-1 (PD-1). However, that does not represent immune exhaustion; rather, these cells exhibit higher proliferation and function, which suggest PD-1 as a marker of activation and functional antigen-specific CD8 T cells in case of DENV infection [41]. Similarly, expansion of the CD4 T cell population occurs during DENV infection, and the magnitude of the CD4 T cell response correlates with disease severity (Figure 1).

Recently, the interactions between DENV and T cells have been explored during the disease course. It has been reported that primary naïve CD4^+^ and CD8^+^ T cells are permissive for DENV infection [42]. Moreover, both cell types support viral replication and secrete viable virus particles. In fact, DENV can infect CD4^+^ and CD8^+^ T cells during acute-phase infection. CD4^+^ and CD8^+^ T cells can be infected by all four serotypes and present similar levels of infectivity. DENV infects and replicates in CD4^+^ and CD8^+^ T cells through interaction with the heparan sulfate moiety [42]. Pre-exposure of DENV to heparin, an inhibitor of host heparin sulfate–virus interactions, limited the virus infectivity in both CD4^+^ and CD8^+^ T cells in a dose-dependent manner. Further, treatment of CD4^+^ and CD8^+^ T cells with heparinase III, a heparin sulfate-cleaving enzyme, reduced DENV infectivity, confirming that heparan sulfate moieties are engaged in DENV binding to T cells. In vitro exposure of healthy peripheral blood mononuclear cells (PBMCs) to all DENV serotypes demonstrated that CD8^+^ T cells are more vulnerable to DENV infection than those of CD4^+^ T cells. A caveat to this is that activated T cells are less susceptible to DENV infection than nonactivated T cells. This was confirmed by the polyclonal stimulation of T cells with anti-CD3/anti-CD28 monoclonal antibodies that induced the expression of CD69 (an early T cell activation marker) on these cells. The susceptibility to infection was determined in both CD69^+^ and CD69^−^ T cells, which revealed that CD69^+^ T cells are less inclined to get infected with DENV in comparison to CD69^−^ cells [42]. Moreover, DENV infection does not induce apoptosis of CD4^+^ and CD8^+^ T cells, a critical step required for viral clearance, suggesting a potential viral escape mechanism leading to disease severity. Other studies reported that DENV infection triggers apoptosis in various parenchymal as well as nonparenchymal cells, including monocytes, dendritic cells (DCs), endothelial cells, and hepatocytes [43,44,45]. Apoptosis of monocytes and DCs influences immune response to infection, while apoptosis of hepatocytes and endothelial cells contributes to hepatic damage and hemorrhagic manifestations in severe dengue cases. Apoptosis of leukocytes and microvascular endothelial cells in pulmonary and intestinal tissue is observed and might be associated with vascular plasma leakage [46].

## 3. Antibody-Dependent Enhancement-Mediated Immunopathogenesis

**Figure 2:**
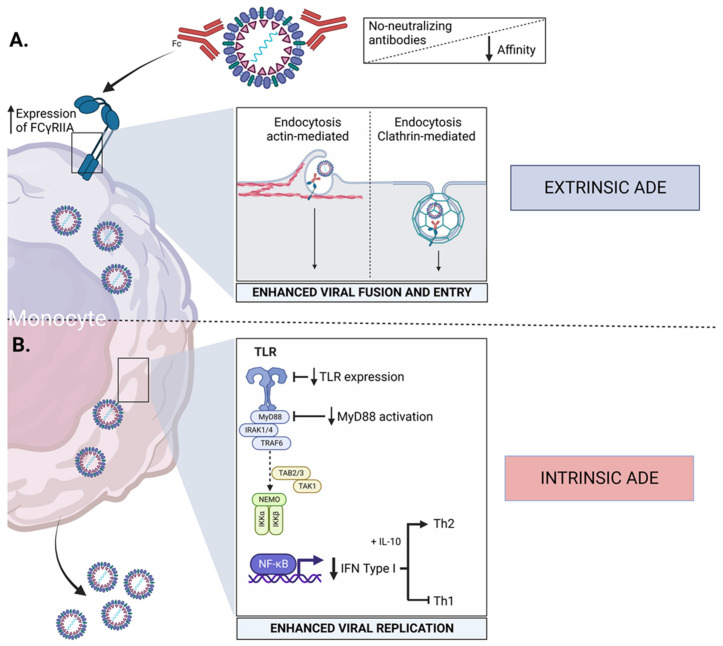
Antibody-dependent enhancement (ADE) in dengue virus infection. ADE is one of the phenomena contributing to the severity of dengue infection during a secondary heterologous infection in a patient with pre-existing non-neutralizing antibodies. The ADE phenomenon has two components: (**A**) Extrinsic ADE, which contributes to the enhancement of virus entry in the susceptible cells via the interaction of the Fcγ of the antibody–virus complex with the Fcγ receptor in the cell membrane, triggering endocytosis (actin- or clathrin-mediated), facilitating the first steps in viral replication. (**B**) Intrinsic ADE, which results in increased viral production by targeting different pathways, including downregulation of TLR signaling, inhibition of type I interferon, and a skewed immune activation favoring Th2 response.

DENV-specific antibodies perform a wide range of functions. These antibodies clear the infection through various mechanisms including restraining the viral binding to cell surface receptors or by inhibiting viral infusion after binding. Neutralizing antibodies mainly target the envelope (E) protein and are directed against almost all the epitopes [47]. Both monocytes and macrophages express the immunoglobulin receptors. The tropism of DENV for these receptors provides an opportunity for DENV-specific antibodies to encourage viral entry, known as antibody-dependent enhancement (ADE) of infection [48]. The phenomenon of ADE contributes to the severe form of the disease and could be itself classified as a type of immunopathology. In ADE of infection, pre-existing antibodies produced from prior DENV infection bind to another DENV-infecting particle in subsequent infection with different DENV serotypes [49]. The antibodies from the previous infection are not efficient in neutralizing the virus in secondary infection. Rather, the antibody–virus complex binds to the Fcγ receptor present on circulating monocytes and causes infection in monocytes, resulting in increased viral replication and higher risk of severe dengue. There are two types of ADE, namely extrinsic and intrinsic [26,50]. The phenomenon of extrinsic ADE occurs externally to mononuclear phagocytes and involves an increased rate of receptor interaction and internalization of the virus–immune complex. Previously, extrinsic factors were considered responsible for the adverse effects of dengue ADE-associated pathogenesis. Later, the intrinsic ADE revealed that it could modulate innate immune effectors by internalized virus–immune complexes inside the infected cells to support increased viral replication and its release [50]. Intrinsic ADE contributes to enhanced virus production by inhibiting type 1 interferon, activating the biosynthesis of IL-10 and favoring a Th2-type immune response [26]. If we compare both extrinsic and intrinsic ADE, the latter has a significant contribution in augmenting dengue replication. During secondary DENV infection, thorough revelation of intrinsic ADE might enhance our understanding of DENV pathogenesis and help in identifying unique therapeutic approaches to overcome the adverse effect of ADE (Figure 2).

Several studies have illustrated that ADE can alter molecular signaling to weaken the host’s antiviral responses. Monocytes, macrophages, and DCs express Fcγ receptors on their surface, and hence presumably are the cell types for ADE under an in vivo setting. In fact, a recent study identified the mechanism to decrease the impact of ADE in the host and provided evidence that antibody-dependent cellular cytotoxicity (ADCC) antibodies are present in the same serum that causes ADE, and these ADCC antibodies are efficient in activating natural killer (NK) cells to fight against ADE [51]. The study further revealed that NK cell degranulation and cytokine production might be associated with lower ADE. Since ADE occurs in the circulation before NK cells are optimally activated, NK cells are not able to control ADE activation, but once activated, these cells can control the progression of the infection. In the setting of ADE, NK cells’ depletion in the PBMCs results in more infection than whole PBMCs, suggesting that NK cells can partially reduce ADE. Moreover, NK cells can suppress newly arising ADE and can control the magnitude of ADE through the process of degranulation and IFN-γ secretion. The neutralization of IFN-γ confirmed that suppression of ADE was weakened in the absence of IFN-γ. ADE development during secondary DENV infection with heterologous serotypes prevents toll-like receptor (TLR) expression and signaling [52]. The presence of negative regulators, including TAF family-associated NF-κB activator (TANK) and sterile-alpha armadillo motif (SARM), contributes to the downregulation of the TLR signaling molecule and expression of TLR-3, 4, and 7 in DENV-infected cells, resulting in the inhibition of IRF1 and IRF3 production [53]. Although extrinsic and intrinsic mechanisms of ADE immunopathogenesis have been related to increased DENV entry into the susceptible cells, this is not always the case in primary macrophages. The ADE-mediated entry of virus particles in primary macrophages induces higher membrane fusion potential within the endosomes, thus increasing virus replication as well as translation [54]. Since viral entry into these cells remains unchanged, the internalized DENV particles remain unrecognized by the endogenous interferon pathway, which allows for enhanced viral replication during the early stages of infection. In addition, intrinsic ADE-mediated upregulation of IL-10 biosynthesis plays a critical role in diminishing the host-mediated innate and adaptive immune response, resulting in viral persistence and leading to disease severity [55]. Hence, understanding the involvement of ADE in DENV pathogenesis is critical and may aid in the development of host-targeting antivirals.

## 4. Cytokine Storm-Mediated Immunopathogenesis

**Figure 3:**
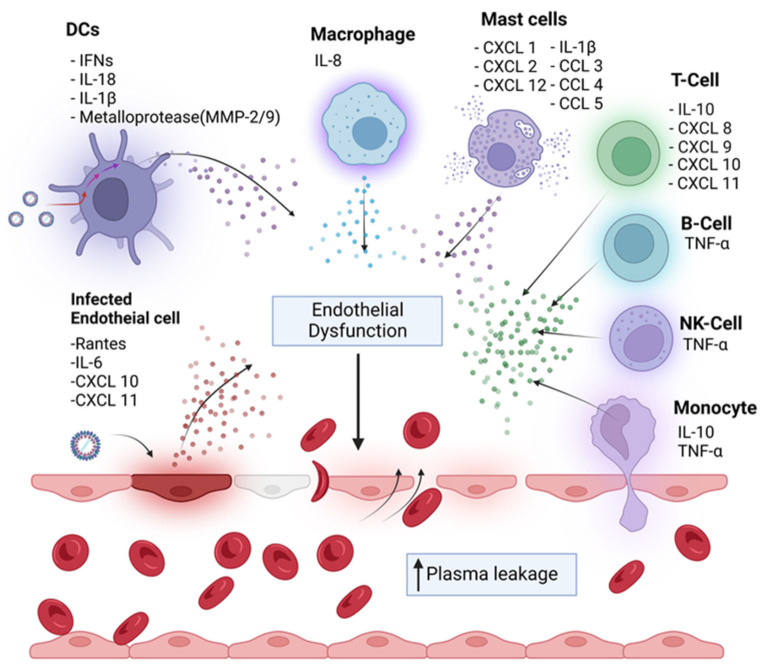
Cytokine storm seen in severe dengue infection. During a secondary heterologous infection, a robust immune response is generated, inducing the production of biological mediators including cytokine, chemokine, and other soluble factors from different immune cells as a consequence of complex interactions between the virus and host factors. These mediators promote vascular permeability resulting in an increase in plasma leakage.

Various proinflammatory, immunoregulatory, and antiviral cytokines are released after the interaction between DENV and host cells. Generally, DCs are known to produce type I interferons; but they can also secrete other molecules and proinflammatory cytokines. Studies have reported that DENV-infected DCs secrete matrix metalloproteinase (MMP)-2 and 9 contribute to increased endothelial monolayer permeability [56,57]. Different DENV proteins, including NS4B and NS5, also stimulate the production of IL-8 by macrophages and endothelial cells [58]. After DENV infection, the release of IL-6, CXCL10, CXCL11, and RANTES from endothelial cells enhances inflammation and vascular permeability, leading to plasma leakage in vivo. Primary infection with DENV alters cell surface receptor expression on human endothelial cells and its responsiveness towards vascular endothelial growth factor-A (VEGF-A); however, these changes were observed in both DENV-infected as well as noninfected cells, suggesting that the endothelial response to DENV is broad and independent of viral-specific cell surface markers. [59]. Indeed, cytokines produced by T cells have pleiotropic effects and are critical in viral elimination, but they also encourage inflammation and enhance vascular permeability contributing to disease severity. Increased T cell activation and cytokine production has been observed during DHF in both primary and secondary DENV infection [60,61]. In fact, infusion of IL-2 or TNF can enhance systemic vascular leakage, which provides evidence for the participation of T cells in the pathogenesis of DHF [62]. In cases of severe dengue, the cascade of immune response mediated by immune effectors leads to disease severity. During DENV infection, most of the immune cells, including monocytes, macrophages, NK, invariant natural killer cells (iNKT), and DENV-specific CD4 and CD8 T cells, secrete huge amounts of TNF-α that contribute to inflammation and enhanced vascular permeability [62]. In vitro studies have confirmed that endothelial cell exposure to TNF-α not only induces vascular permeability but encourages cell death [63]. In addition, TNF-α is involved in the coagulation pathway by inducing the expression of platelet tissue factor. In brief, TNF-α is a critical factor in the pathological process of severe dengue. A study reported higher TNF-α levels in patients with severe illness in comparison to those with milder illness, which further proves the association of TNF-α in the development of severe dengue [64,65]. Considering the crucial role of TNF-α in the pathogenesis of DENV, the blockade of TNF-α can be a potential approach to treat severe dengue infection in humans; however, the safety and efficacy of the TNF-α blocker needs thorough investigation. Interferon receptor gene knock-out mice or mice infected with high doses of either wild-type or mouse-adapted DENV strains revealed that endothelial cell damage was associated with macrophage-secreted TNF-α [66]. Treatment of these models with anti-TNF-α abrogated hemorrhage. A recent study analyzed a panel of 23 cytokines after a dengue outbreak in Taiwan and found that TNF, IFN-γ, IL-1β, IL-2, IL-6, IL-8, IL-10, IL-12p70, IL-17A, macrophage migration inhibitory factor, CD54, CD62E, CD62L, and GM-CSF were elevated while CD106, CD154, IL-4, and IL-33 were downregulated during dengue infection, suggesting the activation of the immune system during dengue, contributing to disease severity, as the dysregulation of certain cytokines has major implications in dengue pathogenesis [67]. The cytokine profile varies during dengue infection and further disease progression. The study further reported that cytokine levels fluctuate immensely in different groups over the days of fever. In fact, IL-10 emerged as a potential diagnostic marker for dengue fever, and CD121b (an IL-1 receptor) was demonstrated to be a predictive marker for severe dengue. During disease progression, the production of inflammatory cytokines, including TNF-α, IL-6, and IL-10, from the DENV-infected cells, induces the expression of adhesion molecules such as CD62E, CD106, and CD62P that lead to inflammation, endothelial damage, and plasma leakage. Adhesion molecules such as CD54, CD106, CD62E, CD62L, CD62P, and CD154 fluctuated at different days of fever and normalized during the recovery phase, whereas GM-CSF, IL-2, IL-6, and IL-8 remained continuously high, especially during the critical phase of the disease [67]. Several pieces of evidence suggest the change in cytokine pattern during dengue infection and support the cytokine storm theory in these patients. In addition, a shift from the Th1-type immune response to Th2-type response was observed during disease progression to severe cases of DHF [68]. Patients with DHF displayed increased serum levels of IL-4, IL-6, and IL-10, mainly in more severe forms. Conversely, IFN-γ and IL-2 were highly elevated during dengue fever and were low in severe DHF. IL-2, IL-6, IFN-γ, and TNF-α were elevated during the early phase of infection, while IL-4 and IL-10 were elevated during days 4–8 of illness. However, the data are contradictory between different studies, which could be associated with several factors, including the timing of sample collection, sample processing, and importantly, the difference in the study cohort. However, the overall data demonstrate cytokine-mediated pathogenesis in DENV-infected patients (Figure 3).

## 5. Infection of Immune Cells with DENV Contribute to the Pathogenesis

DENV can infect several types of parenchymal and nonparenchymal cells, including epithelial and endothelial cells, hepatocytes, muscle cells, DCs, monocytes, macrophages, mast cells, and B and T cells. The presence of DENV antigens such as E protein and NS3 have been detected in different tissues, including skin, liver, spleen, lymph node, kidney, bone marrow, lungs, thymus, and brain [69,70,71,72]. However, these viral particles have not always been isolated from these organs except from liver and PBMCs, suggesting that the immune cells and the liver may be the key targets for DENV replication during DENV infection [73].

In terms of immune cells, DCs, monocytes, macrophages, and B cells are the primary targets for DENV infection. Several studies indicated that C-type lectins, including dendritic cell-specific intercellular adhesion molecule-3 grabbing non-integrin (DC-SIGN) and C-type lectin domain family 5, member A (CLEC5A), which are present on the surface of DCs and macrophages, act as principal receptors for DENV [74,75,76]. Other DC receptors, including mannose receptors, langerins, Fc-receptors, T cell immunoglobulin, and mucin domain-containing protein (TIM)-3, TIM4, and AXL, also act as DENV receptors [77,78,79]. However, different receptors possess different functions. DC-SIGN functions as a viral attachment factor, whereas CLEA5A induces the production of antiviral and proinflammatory cytokines, suggesting that C-type lectin may act as a cognate receptor for DENV virions. DENV also infects mast cells in the skin and activates various cytokines and chemokines and increases degranulation in mast cells, suggesting a potential involvement of mast cells in the development of severe dengue and subsequently to vascular leakage [80,81]. After dissemination from the skin, macrophages in the lymphoid and nonlymphoid cells act as primary reservoirs for DENV. Dengue viruses use a group of cell surface receptors, including mannose receptors CD205, heat shock proteins (HSP70/HSP90), CD14-associated protein, CD300a, TIM4, PD1, and Fc receptors (FcγRI and FcγRII) to infect monocytes and macrophages [82]. Binding to Fc receptors appears to play an important role in secondary DENV infection.

Dengue virus can also infect B cells [42]. A humanized mouse experiment confirmed DENV infection in B cells, subsequently stimulating the production of proinflammatory cytokines [83,84]. DENV infection influences B cell phenotypes, shown by increased frequencies of CD19^+^ B cells in dengue patients [85]. Similarly, frequencies of plasmablasts and plasma cells are massively increased during the acute phase of DENV infection [86]. The activation of B cells and development of plasma cells have been observed more frequently in hospitalized dengue patients than asymptomatic DENV-infected patients. A study by Upasani et al. reported that B cells from DENV-infected patients support viral replication of both clinically derived as well as laboratory-adapted DENV strains in ex vivo and in vitro conditions by attaching to CD300a, a phosphatidylserine receptor, for viral entry into B cells. CD300a recognizes phosphatidylserine and phosphatidylethanolamine present in viral envelopes derived from the lipid bilayer of the host plasma membrane [85]. Although DENV infection is not fully inhibited, the blockade of CD300a on B cells decreases the infection in a concentration-dependent manner, which further confirms that CD300a acts as an attachment and entry receptor for DENV infection in B cells. Furthermore, DENV infection induces B cell proliferation in dengue patients in vivo and plasmablasts and plasma cell formation in vitro. The B cell response to DENV infection may be responsible in the pathogenesis of dengue.

## 6. Contribution of Exosomes during Dengue Infection

Exosomes are small membrane vesicles secreted to the extracellular space by various cell types. Exosomes released from virus-infected cells are accountable for viral transmission through cell-to-cell communication. Since viral infections stimulate the secretion of exosomes, it is logical to assume that DENV infection could also induce exosome release in dengue-infected patients [87]. The release of exosomes from DENV infected cell line C6/36 (cell line often used for DENV amplification) has been reported [88]. Dengue infection alters the composition of exosomes secreted from the infected cells. These exosomes carry a full-length DENV genome with other proteins and transmit viral particles to healthy cells [89]. In addition, exosomes released from DENV-infected cells contain LC3 II, an autophagy marker that protects the virus from anti-dengue neutralizing antibodies [90]. DENV infection induces the activation of platelets through C-type lectin-like receptor 2 (CLEC2) and increases the secretion of exosomes as well as microvesicles [91]. These exosomes further increase the formation of neutrophil extracellular traps and derive enormous cytokine production from macrophages and neutrophils. Platelet-derived microparticles also carry IL-1β, which is strongly associated with increased vascular permeability. Extracellular vesicles represent a new mechanism of dengue pathogenesis. CD9 exosomes carry ALIX with PrM/E viral proteins released from DENV-infected mosquito cells and can infect naïve mosquito cells [88]. In addition, previous studies have detected DENV envelope proteins in exosomes. Extracellular vesicles released by monocyte-derived dendritic cells (moDCs) infected with DENV-3-5532 exhibit messenger RNAs (mRNAs) for CXCR4, MIF, IL-17A, and IL-18 [92]. These molecules are involved in disease severity in DENV-infected patients along with endothelial cells and platelet activation and mediate plasma leakage and dengue shock syndrome. Moreover, microRNA present in moDCs infected with DENV-3-5532 regulate transforming growth factor-β (TGF-β), Erb, MAP kinases, phosphatidylinositol-3-kinase/Serine-threonine kinase (PI3K/AKT) pathways, which could promote DC apoptosis and a proinflammatory environment during DENV-3-5532 infection [93].

**Figure 4:**
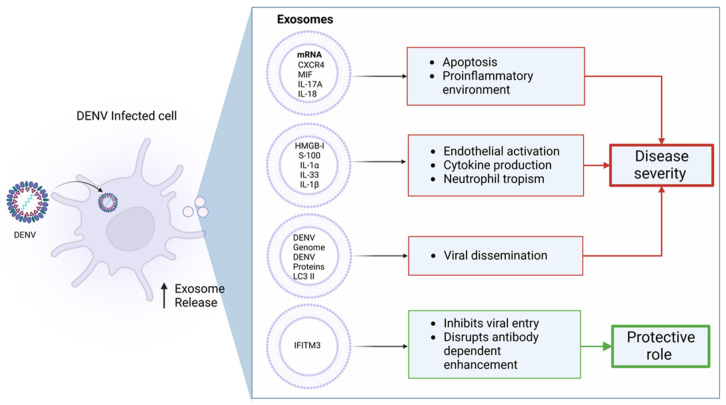
Exosomes during dengue virus infection. DENV infection induces the secretion of small membrane vesicles (known as exosomes) from infected cells to the extracellular space. These exosomes perform different functions such as the induction of cytokine production, apoptosis, endothelial cell activation, viral dissemination, inhibition of viral entry, and interference with ADE phenomenon depending on its content and cell origin, contributing to disease severity or protection against infection.

Exosomes are not only involved in DENV transmission but also play a protective role. They carry interferon-induced transmembrane protein 3 (IFITM3), which transfers the antiviral response between different cells during DENV infection and suppresses the entry of the enveloped virus [94]. A cellular model of DENV infection reveals that exosomal IFITM3 inhibits DENV entry under different conditions, including at baseline and interferon-induced conditions. Another study found that IFTIM proteins have the potential to interfere with the ADE phenomenon during secondary DENV infection. IFTIM can limit both direct and ADE-associated secondary DENV infection, suggesting the dual role of exosomes during DENV infection (Figure 4).

Endothelial activation is a well-recognized process during DENV infection. A study by Velandia-Romero and colleagues demonstrated that the extracellular vesicles derived from DENV-infected U937 macrophage cell line transport proteins and microRNA encourage early changes in the endothelial physiology, resulting in activation of the vesicles and provoking a defensive pathway against damage during the first stages of the disease [95]. The treatment of extracellular vesicles with IFN-α can restrict DENV3-5532 infection in PBMCs, suggesting that extracellular vesicles could communicate between cells to share defensive signals to inhibit viral replication and thus reduce viral replication [92]. The process of endothelial activation is performed by the secretome of extracellular vesicles, including HMGB-1, S-100, IL-1α, and IL-33, which introduces new players involved in cell response during DENV infection. Extracellular vesicles released from DENV-infected or activated cells are quickly released and circulate in the blood, stimulate the endothelium as well as other immune cells, and contribute to the development of the first protective proinflammatory response during the infection.

## 7. Role of Interferon-Stimulated Genes during Dengue Infection

The role of several interferon-stimulated genes (ISGs) in DENV replication has been reported [96]. These genes target DENV at multiple stages of the viral replication cycle. Several of these genes are well-known inhibitors of viral replication, while the majority of them remain uncharacterized with respect to their antiviral potential. DENV infection induces the expression of various ISGs, including tripartite motif (TRIM) protein-encoding gene (TRIM69), LGALS3BP, C190ORF66, DDX60, FBXO15, and HELZ2 [97]. mRNA as well as protein expression of TRIM69 was elevated in DENV-2 infection in a virus dose-dependent manner and was found to be upregulated in A549, human umbilical vascular endothelium (HUVEC) cells, and DENV-2-infected human and mice PBMC samples. Overexpression of TRIM69 inhibited DENV replication and induced antiviral activity. The DENV-2-infected mice model confirmed that TRIM69 directly interacts with DENV nonstructural protein NS3, mediating its polyubiquitination and degradation, controlling DENV infection [97]. ISGs represent the major effector mechanism of innate immunity and help in developing a rapid adaptive immune response to clear the infection. ISG15 is efficient in controlling viral replication by facilitating the cellular antiviral response, as shown in an RNA interference study where the silencing of IGS15 by siRNA in RAW264.7 cells increased the DENV load by 2.1-fold compared to those of controls, confirming its potent anti-DENV role in an in vitro model [98]. Interferon, an antiviral cytokine, mediates its function through the induction of hundreds of ISGs. Amongst the three main interferon (IFN) families, type I (IFNα/β) and III (IFNλ) are primarily considered antiviral IFNs, while type II (IFNγ) has well-defined antiviral effects. IFNs are transcriptionally activated by a sequence of events through viral sensors, adaptor proteins, kinases, and transcription factors influencing several host processes to generate antiviral effects [99]. IFN-I stimulates the expression of various genes in infected as well as noninfected cells after direct recognition of DENV-associated components such as DENV-related antigens, ssRNA, and dsRNA by toll-like receptors [15]. Interaction between the viral and host factors leads to the activation of innate immune cells that secrete numerous components to control viral infection. Although all mononuclear phagocytes are capable of producing type I IFNs, dendritic cells remain the primary source. The binding of IFN-I to its respective receptor leads to the activation of tyrosine kinases, janus kinase 1, and tyrosine kinase 2, which phosphorylate STAT1 and STAT 2 to form a heterodimer that further associates with IRF-9 and forms a multimeric complex, IFN-stimulated gene factor 3. This complex then translocates to the nucleus-deriving transcription of ISGs, leading to the induction of antiviral immunity by the production of antiviral cytokines [100]. IFN blocks DENV infection via the suppression of DENV RNA translation through a protein kinase R (PKR) mechanism [101]. Knockdown of PKR by short interfering RNAs downregulates IFN synthesis through the RIG-1/IPS-1 pathway in DENV-infected human lung epithelial cells, suggesting the critical role of PKR in IFN synthesis [101]. Before DENV infection, in vitro treatment of human HepG2 cells with IFN-α/β or IFN-γ protects these cells from viral replication while treatment performed after DENV infection does not control viral replication, indicating that DENV has developed an antagonist activity against the IFN-I-mediated immune response in host-infected cells [102]. Several lines of evidence indicate that DENV has developed resistance to IFN-I (IFNα/β), which might be associated with the increased expression of Ubiquitin-specific protease-18 (USP18), a negative regulator of IFNα/β signaling, found in DENV-2-infected Hela cells and patients’ blood [103]. Overexpression of USP18 promoted DENV-2 replication, while its silencing abrogated the replication and increased the anti-DENV-2 specific IFN-α through the activation of the IFN-α-mediated Jak/STAT signaling pathway, as shown by the increased expression of p-STAT1/p-STAT2 and the elevated expression of some ISGs. Overall, USP18, stimulated by DENV-2 infection, is a critical host factor exploited by DENV-2 to confer IFN-α antagonism [103].

## 8. Immune Escape Mechanism of DENV Contributes to Disease Severity

It is a well-known fact that dengue virus takes over the host cellular machinery to complete its life cycle. This leads to the activation of cellular immune signaling, which in turn competes against infection. DENV antagonizes several challenges at each step of its life cycle, starting from the virus entry to the release of mature virions. Moreover, DENV inertly hides to escape immune surveillance and directly targets immune mediators to block antiviral signal transduction. Innate immunity acts as the first line of defense against viral infections, where type I interferons are one of the major players. Many viruses evade innate immunity and develop infection in the host. Numerous studies have shown that viruses including DENV inhibit type I IFN production to evade the host immune response [20]. Several DENV proteins are involved in the inhibition of type I IFN signaling in the infected cells, leading to the abrogation of IFN genes and thus restricting antiviral function [99]. Pathogen-associated molecular patterns (PAMPs), including TLRs (TLR3/7/9) present on different APCs, recognize the viral particles and get activated [104,105]. Cytosolic receptors, including RIG-1/MDA-5, also recognize the viral particles and trigger several pathways, resulting in the production of cytokines and chemokines that control viral infection [106]. Although DENV is a weak inducer of type I IFN after infection in DCs, it still impairs the type I IFN pathway and decreases the ability of DCs to generate a Th1-type immune response, which enables viral persistence [107]. DENV can overcome host innate immunity and can infect via two mechanisms: first by evading the collaboration between PAMPs and PRRs, and the other by inhibiting various steps of the innate immune response through the expression of inhibitory molecules, which directly blocks the intracellular pathways required for type I IFN production and signaling [108]. Moreover, DENV NS4A potentially disturbs the induction of IFN by targeting mitochondrial antiviral signaling protein (MAVS) [109]. NS4A binds to MAVS and inhibits the binding of MAVS with RIG-1, and thus prevents IFN production. In addition, NS4A associates with the N-terminal CARD-like (CL) domain and C-terminal transmembrane domains of MAVS, as well as the third transmembrane domain of NS4A, which is indispensable for binding to MAVS, suggesting that NS4A is involved in DENV immune evasion through the inhibition of the MAVS-mediated cellular response. Conserved viral RNA conformation is another DENV escape strategy. DENV genomic RNA is capped at the 5′ end, similar to cellular mRNA, which is post-transcriptionally capped at the 5′ end, encompassing one N-7 methylguanosine and one or two 2′-O methylguanosine. Hence, viral RNA deficient in 2′-O-methylation will be recognized as non-self RNA, whereas DENV lacking 2′-O methyltransferase activity enables an early innate immune response in the host cells and replicates with a lower viral load than the wild type, and thus escapes and stays under the radar in host cells [110].

## 9. Dengue Serotypes Contribute to Disease Severity

The clinical spectrum of DENV ranges from asymptomatic to severe presentation; however, the determinants of severity are not well defined. Prospects regarding pathogenesis mainly emphasize interactions between the virus and its host. Additionally, the genetic background of the host, certain viral strains, and immune response to previous dengue infection predispose to worse outcomes [111,112]. It has been reported that DENV serotypes and their structural peculiarities also contribute to pathogenesis [113]. As viral genetic factors govern its virulence and the magnitude of viral replication determine disease severity, serotypes with higher replication capacity trigger higher antibody production and may be associated with more severe outcomes [114]. A study of 485 DENV cases in a locality in Brazil highlighted the influence of DENV serotypes on clinical manifestations and outcomes, of which 6.6% presented severe disease, which mostly belonged to the DENV-2 serotype (32.3%) and less in DENV-1 (4.5%) and DENV-4 (6.4%), suggesting that early serotype identification could be beneficial in preventing a growing number of severe outcomes, especially during dengue outbreaks, by predicting the health support required for early diagnosis and interventions [115]. Other studies also observed similar findings with an increased proportion of severe outcomes, such as DHF and DSS in infections caused by DENV-2 [116,117,118]. DENV-2 appears to be a determinant factor for the emergence of severe dengue in different global regions and epidemics with increased hemorrhagic cases in regions where DENV-2 is the leading serotype; however, the mechanism of virulence is not well defined. One of the possible factors is the stimulatory effect of DENV-2 on nitric oxide production, instigating toxic and inflammatory effects, prompting apoptosis in host cells [119]. Another contributing factor to the increased pathogenicity in DENV-2 infections is its higher viral replication, leading to high viral load [120]. Prior reports of DENV in Thai children have reported that secondary DENV-2 is more likely to result in severe disease compared with other serotypes [121]. Other data from a Nicaraguan cohort further confirmed that 29% of DENV-2 hospitalized cases emerged as DHF/DSS in the 2005/2006 season, while this percentage upsurged to 63% across further seasons [113]. Overall, these studies indicate that DENV-2 appears to be associated with severe outcomes. However, more recent data involving a Vietnamese large cohort found that having a higher plasma viremia during the febrile phase was associated with adverse outcomes such as vascular leakage, severe dengue, and subsequent hospitalization regardless of infection serotype or host immune status [122]. Nevertheless, DENV-2 carries the greatest risk of adverse outcomes in this population as well, despite manifesting lower daily viremia compared to other serotypes [122].

## 10. Conclusions

The pathogenesis of dengue infection is attributable to both host and viral factors, including nonstructural proteins, subgenomic RNA, genome variation, immune cells and its soluble factors, antibody-dependent enhancement, and the presence of cross-reactive T cells. Although gaps remain, progress in the understanding of the immune mechanisms associated with both viral clearance as well as disease severity may hopefully translate to the development of robustly protective vaccines against DENV. Moreover, to date there are no specific treatment options available for these patients, suggesting that regardless of the advancements in our current knowledge of DENV infection, we are still not up to date in understanding dengue-associated immunopathogenesis, and further investigations are warranted for in-depth characterization of innate and adaptive immune cells and their association with disease severity.
